# Tularemia in Pregnant Woman, Serbia, 2018

**DOI:** 10.3201/eid2904.221318

**Published:** 2023-04

**Authors:** Milena Saranovic, Marija Milic, Ivan Radic, Natasa Katanic, Mirjana Vujacic, Milos Gasic, Ivan Bogosavljevic

**Affiliations:** University of Pristina, (temporarily in) Kosovska Mitrovica, Serbia (M. Saranovic, M. Milic, I. Radic, N. Katanic, M. Gasic, I. Bogosavljevic);; Clinic for Infectious Disease, Health Center, Kosovska Mitrovica (N. Katanic, M. Vujacic)

**Keywords:** tularemia, pregnancy, Francisella tularensis, bacteria, zoonoses, vector-borne diseases, Serbia, Europe

## Abstract

Tularemia was diagnosed for a 33-year-old pregnant woman in Serbia after a swollen neck lymph node was detected at gestation week 18. Gentamicin was administered parenterally (120 mg/d for 7 d); the pregnancy continued with no complications and a healthy newborn was delivered. Treatment of tularemia optimizes maternal and infant outcomes.

Tularemia is a systemic, potentially serious zoonotic disease caused by *Francisella tularensis* bacteria (*1*). Interhuman transmission of tularemia has not been reported. Transmission is primarily by Ixodidae ticks (*2*,*3*); the second most frequent vectors are mosquitoes in restricted areas (e.g., Sweden and Finland) (*4*). Contact with live infected animals can be a source of human infection. Many tularemia cases also occur through contact with contaminated aquatic environments (e.g., swimming, canyoning, fishing) (*5*,*6*). Incubation period is 1–10 days, and clinical signs depend on the route of infection. Direct contact with animals or tick bites leads to a skin lesion with satellite lymphadenopathy. Ingesting contaminated food or drinking contaminated water leads to pharyngitis and cervical lymphadenopathy. Clinical signs are ulceroglandular disease with cutaneous ulcerations and marked lymphadenopathy; glandular disease with no ulcers but marked lymphadenopathy; oculoglandular disease with preauricular lymphadenopathy and conjunctivitis; oropharyngeal disease with pharyngitis and cervical lymphadenopathy; gastrointestinal disease with vomiting, abdominal discomfort, and diarrhea; respiratory disease (pneumonia or pleuritis); and typhoid tularemia (fever without early signs/symptoms) (*7*). If not treated adequately, tularemia can spread to the lungs, pleura, gastrointestinal tract, or central nervous system (*8*,*9*).

There are 2 subspecies of *F. tularensis*: type A, which is almost completely restricted to North America, and type B, which is found in Europe (*1*). Type A strains are highly virulent. Before the advent of effective antimicrobial drugs, mortality rate for both types was 5%–10% (*10*). Current mortality rates are 2%–3% for type A and <1% for type B tularemia but vary according to type of infection and *F*. *tularensis* genotype. Mortality rates among patients with bilateral type A acute pneumonia are >30% (*7*).

After 2000, the annual incidence rate of tularemia in Europe was highest in Kosovo (>5 cases/100,000 population), followed by Sweden, Finland, Slovakia, Czech Republic, Norway, Serbia, Hungary, Bulgaria, and Croatia (*1*). Tularemia is more common in men than in women and extremely rare in pregnant women (*2*,*3*). To date, at least 12 cases of tularemia during pregnancy have been described in the literature (Appendix). We report the case of a pregnant woman with tularemia in Serbia.

## The Case

In 2018, a 33-year-old woman in the second trimester of pregnancy (18 weeks’ gestation) was referred to the Gynecology Department of the Policlinic for Students’ Healthcare, University of Pristina, for a painless lymphadenopathy on the left side of her neck. The woman denied having a fever or other signs/symptoms of infection. She lived in the urban area of Kosovska Mitrovica and reported no contact with known reservoirs of *F. tularensis*, although she did mention having gone for a walk and spending some time in natural areas, but not swimming, a few days before symptom onset. She denied drinking nonpotable water (e.g., spring water) and confirmed that she drank only bottled water.

Three months before pregnancy, cervical screening (including smear test and microbiology) detected no abnormalities. She had no previous pregnancies or remarkable medical or surgical history. During week 7 of gestation, a viable, eutopic, singleton pregnancy was confirmed, and at week 12, gynecologic and ultrasonography examinations and double marker, blood, urine, and biochemical testing were performed. Fetal growth corresponded with the length of the amenorrhea, and all results were within the normal range for pregnancy. Double marker testing indicated low risk for trisomies. The patient was asymptomatic. The next appointment was at week 16 of gestation, when uneventful pregnancy with constant growth velocity was confirmed.

The painless lymphadenopathy was detected at week 18. The lymph node was mobile, ≈2 cm in diameter, and painless. Blood analysis results, including leukocytes, were within reference limits and showed no evidence of bacteremia. Nose and throat swab sample cultures showed no microbial growth.

For biopsy of the swollen lymph node, we referred the patient to the Clinic for Otorhinolaryngology and Maxillofacial Surgery at the Clinical Centre of Serbia, University of Belgrade. At the time of the transfer, she was 19 weeks pregnant, febrile, but clinically stable. Fetal growth corresponded to gestational age. Immunologic analyses, blood count, and peripheral blood smear performed by the hematologist ruled out blood disorders and systemic diseases (antinuclear antibodies were negative). Serologic analyses excluded infection caused by Epstein-Barr virus, cytomegalovirus, HIV, herpes simplex virus, and hepatitis B and C viruses. Toxoplasmosis and brucellosis were excluded by relevant testing.

The enlarged neck node was excised with local anesthesia at week 19 of gestation. Histopathologic findings confirmed histolytic necrotizing lymphadenitis (also called Kikuchi-Fujimoto disease), which can be induced by an infectious disease. Serum was sent to SYNLAB (Augsburg, Germany) for *F. tularensis* testing, and after 2 weeks, enzyme immunoassay showed increased IgM against *F. tularensis* (16.1 U/mL, cutoff<10 U/mL) but not IgG, which correlated with the clinical signs and indicated acute infection.

The patient was hospitalized for 2 weeks; she was febrile for 2 days (temperature not >39°C) and treated symptomatically. Ultrasonography for fetal anomaly performed at the Institute for Gynaecology and Obstetrics “Visegradska,” Belgrade, detected no abnormality, and fetal growth velocity was maintained. Blood analysis remained within reference limits, with no evidence of bacteremia. After receiving gentamicin (120 mg intramuscularly 1×/d) for 7 days, the patient was discharged at week 22 of gestation.

At term, the woman vaginally delivered a healthy boy, weighing 3,860 g, with Apgar scores of 9 at 1 minute and 10 at 5 minutes. Histopathologic examination of the placenta revealed no evidence of pathology. Newborn hearing screening on day 3 was normal bilaterally. At examinations performed 1 and 4 months after delivery, both mother and baby were healthy, with no complications, and all blood test results were within reference limits. The newborn was not tested further for infectious diseases.

## Conclusions

According to the available literature, tularemia in pregnancy is rare; at least 12 cases have been described (Appendix). Although gentamicin, ciprofloxacin, and doxycycline are not recommended as the first-line treatment for pregnant women, the World Health Organization recommends ciprofloxacin and gentamicin for treatment of tularemia during pregnancy (*11*–*14*). Use of gentamicin, ciprofloxacin, and doxycycline is not associated with increased risk for birth defects, stillbirth, premature birth, or low birth weight (*12*,*13*). A previous case indicated that during pregnancy, dacryocystitis and abscess on the neck with pharyngitis did not affect the pregnancy outcome after surgical and antimicrobial treatment (*15*). The gradient of symptoms and clinical manifestations of tularemia in pregnant women differ and depend on the mode of transmission of the infection, the amount and virulence of the pathogen, and the host immunity. Mild clinical signs/symptoms can be expected in healthy young pregnant women without other comorbidities who acquire infection in the second or third trimester, as did the patient we report.

Tularemia is extremely rare during pregnancy; mild disease can appear during the second and third trimesters, and adequate treatment can be administered without consequences to the fetus. Therefore, in a pregnant woman with lymphadenopathy, with or without signs/symptoms, infections such as tularemia should be considered in the differential diagnosis, especially in areas where tularemia still circulates at a high incidence rate. To optimize maternal and infant outcomes, tularemia can and should be adequately and effectively treated during pregnancy.

Appendix Additional information about tularemia in pregnant women.
