# Supplementary material for: Tularemia in Pregnant Woman, Serbia, 2018
Source: Emerg Infect Dis. 2023 Apr;29(4):806–8. doi: 10.3201/eid2904.221318 (PMC10045689; doi:10.3201/eid2904.221318)
Supplement: Appendix — Additional information about tularemia in pregnant women. [file 22-1318-Techapp-s1.pdf]

Article DOI: https://doi.org/10.3201/eid2904.221318

EID cannot ensure accessibility for supplementary materials supplied by authors. Readers who have difficulty accessing supplementary content should contact the authors for assistance.

Tularemia in Pregnant Woman, Serbia, 2018

Appendix

In the available literature, tularemia in pregnancy is very rare; 12 cases have been described in the literature: 2 in the United States (*1–3*), 2 in France (*4–6*), and 8 in Turkey (*4*,*6*,*7*). Including the case reported here, all pregnant women who accepted treatment (gentamicin, azithromycin, and amoxicillin/clavulanic acid) and 2 who refused treatment gave birth to a healthy child with no complications; 2 who refused treatment had a spontaneous abortion and 1 had a premature birth. Analyzing cases of tularemia in pregnancy, the outcome of pregnancy without complications and the birth of a healthy child was more common in patients for whom tularemia was diagnosed in the second and third trimesters (*3–9*).

References

1. Centers for Disease Control and Prevention. Key facts about tularemia [2023 Mar 10]]. https://emergency.cdc.gov/agent/tularemia/facts.asp

2. Djordjevic Spasic M. Immunodiagnostic and molecular methods in evaluation of the therapy efficiency on patients suffering from tularemia [PhD thesis]. Nis, Serbia: University of Nis; 2014.

3. Bricker D. Tularemia infection during pregnancy. Am J Nurs. 1931;31:979–82.

4. Charles P, Stumpf P, Buffet P, Hot A, Lecuit M, Dupont B, et al. Two unusual glandular presentations of tick-borne tularemia. Med Mal Infect. 2008;38:159–61. PubMed https://doi.org/10.1016/j.medmal.2007.11.005

5. Dentan C, Pavese P, Pelloux I, Boisset S, Brion JP, Stahl JP, et al. Treatment of tularemia in pregnant woman, France. Emerg Infect Dis. 2013;19:996–8. PubMed https://doi.org/10.3201/eid1906.130138

6. Ata N, Kılıç S, Övet G, Alataş N, Çelebi B. Tularemia during pregnancy. Infection. 2013;41:753–6. PubMed https://doi.org/10.1007/s15010-013-0456-5

7. Yeşilyurt M, Kiliç S, Çelebi B, Gül S. Tularemia during pregnancy: report of four cases. Scand J Infect Dis. 2013;45:324–8. PubMed https://doi.org/10.3109/00365548.2012.720027

8. Dudley PB, Don CW. Tularemia and pregnancy: report of a case. J Am Med Assoc. 1936;107:577–8. https://doi.org/10.1001/jama.1936.92770340001007

9. Yilmaz GR, Guven T, Guner R, Kilic S, Gulen TA, Eser FC, et al. Tularemia during pregnancy: three cases. Vector Borne Zoonotic Dis. 2014;14:171–3. PubMed https://doi.org/10.1089/vbz.2013.1406
